# Epigenetic Control of Cold Stress Tolerance in Plants: Emerging Mechanisms and Applications for Crop Improvement

**DOI:** 10.3390/ijms27146454

**Published:** 2026-07-20

**Authors:** Lixia Sun, Liting Yang, Guanqing Wu, Muhammad Qasim Shahid, Ruziyev Farid Ashurovich, Faheem Shehzad Baloch, Muhammad Azhar Nadeem, Fozia Ghouri

**Affiliations:** 1 State Key Laboratory for Conservation and Utilization of Subtropical Agro-Bioresources, South China Agricultural University, Guangzhou 510642, China; slx@stu.scau.edu.cn (L.S.); yangliting@stu.scau.edu.cn (L.Y.); wuguanqing@stu.scau.edu.cn (G.W.); qasim@scau.edu.cn (M.Q.S.); 2 Guangdong Laboratory for Lingnan Modern Agriculture, Guangzhou 510642, China; 3 Department of Genetics, Samarkand State University Named After Sharof Rashidov, Samarkand 140104, Uzbekistan; f.ruziyev1985@gmail.com (R.F.A.); balochfaheem13@gmail.com (F.S.B.); 4 Department of Biotechnology, Faculty of Science, Mersin University, Yenişehir, Mersin 33343, Türkiye; azharjoiya22@gmail.com

**Keywords:** cold stress, epigenetic regulation, DNA methyltransferases, histone modifications, C-repeat binding factor *(CBF)*

## Abstract

Low temperature is an abiotic stress factor that affects plant development and geographic expansion, resulting in significant economic losses in worldwide food production each year. Through evolution, plants have developed intricate adaptation systems, with epigenetic regulation—modifying gene expression without altering DNA sequences—being pivotal in the cold stress response and memory formation. This review provides a systematic overview of the role of the main epigenetic mechanisms, including histone modifications, DNA methylation, non-coding RNA regulation, and chromatin remodeling, in plant cold stress responses. The article summarized research on plants, including model species *Arabidopsis thaliana* and rice, examined epigenetically mediated cold-stress memory and transgenerational epigenetic inheritance, and analyzed synergistic interactions among regulatory pathways. Finally, by integrating recent advances, the study identifies scientific challenges and research bottlenecks in this field and outlines future research directions and application prospects in cold-resistant crop breeding. Finally, by integrating recent advances, the study identifies scientific challenges and research bottlenecks in this field, outlines future research directions and application prospects for cold-resistant crop breeding, and provides references for further elucidating the molecular mechanisms of plant cold adaptation and for developing new cold-resistant crop varieties. These outcomes offer references for further elucidating the molecular mechanisms of plant cold adaptation and developing new cold-resistant crop varieties.

## 1. Introduction

**Figure 1:**
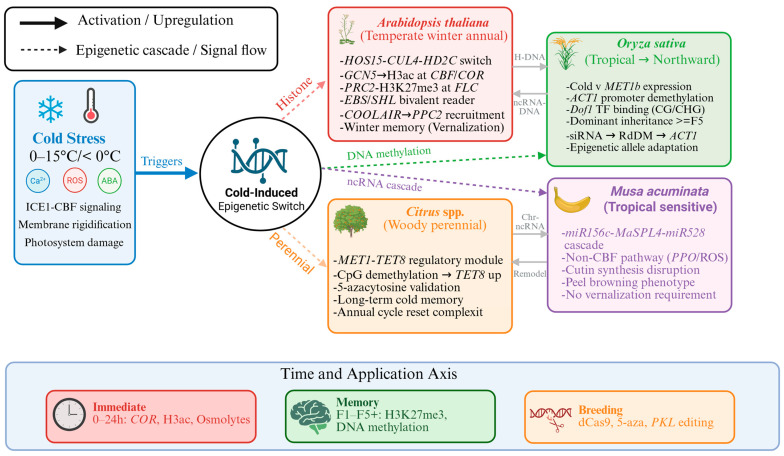
Species-specific epigenetic cascades and transgenerational cold memory in plants. Cold stress triggers Ca^2+^/ROS/ABA signaling and a central epigenetic switch, diverging into four species-specific strategies. *Arabidopsis thaliana* (top left) employs the *HOS15*-*CUL4*-HD2C switch and *GCN5*-mediated H3ac to activate *CBF*/*COR* genes, while PRC2-H3K27me3 and *COOLAIR* silence *FLC* for winter memory. Rice (top right) undergoes *MET1b* inhibition and *ACT1* promoter demethylation (CG/CHG), enabling *Dof1* binding and dominant inheritance (≥F5). Citrus (bottom left) utilizes the *MET1*-*TET8* module; CpG demethylation upregulates *TET8*, validated by 5-azacytosine. Banana (bottom right) operates a non-*CBF miR156c*-*MaSPL4*-*miR528* cascade disrupting cutin synthesis. The bottom axis depicts three temporal layers: immediate response (0–24 h), transgenerational memory (F1–F5+), and breeding applications (dCas9, 5-aza, *PKL* editing). Solid arrows, activation; dashed arrows, epigenetic signal flow.

According to different temperatures, cold stress can be divided into chilling temperatures (0–15 °C) and freezing temperatures (below 0 °C) [1,2]. Cold stress can affect plant development, thereby influencing yield. From a natural ecological perspective, low temperatures determine the geographical boundaries of plant communities. Temperate plants have evolved cold-acclimation mechanisms to adapt to seasonal changes, whereas tropical and subtropical plants, such as bananas and rice, exhibit extreme sensitivity to low temperatures [3,4,5]. After plants are first exposed to temperatures below freezing, their frost resistance is enhanced [6,7]. After the low-temperature signal is triggered, it can quickly induce the corresponding genes, promoting the accumulation of osmotic substances and antifreeze proteins, which helps the process of cold acclimation [8]. Cold stress damages plants by destroying cell membranes [9]. Pores appear in the membrane structure, leading to increased permeability [10]. Intracellular electrolyte leakage and efflux of cellular ions thus damage the structure of the cell membrane [11]. Changes in osmotic pressure can induce the efflux of water from the cell through the plasma membrane into the intercellular space, leading to plasmolysis [12]. Persistent water loss causes cell volume to shrink and accelerates the destruction of cell structures. Water in the intercellular spaces forms ice crystals, which expand in volume, exert mechanical pressure on cells, and may also cause cell wall rupture. As intracellular water is lost, the concentration of solutes inside the cell relatively increases, sugar content in vacuoles changes, cytoplasmic homeostasis is disrupted, and high concentrations of ions such as K^+^ and Ca^2+^ may interfere with protein activity [13]. Organelles such as chloroplasts, vacuoles, and mitochondria are all damaged. The structural damage of chloroplasts further affects photosynthesis and systemic processes [14,15]. The light-dependent electron transfer chain is inhibited, resulting in excess light energy, leading to photoinhibition [16]. The cell nucleus is relatively stable in the early stages. However, under long-term stress, the chromatin may aggregate, and the nuclear membrane may collapse or rupture. The types of responses to cold stress include various functional mechanisms such as transcription factors, the inducer of C-repeat binding factor, and protein kinases [7,8,17,18]. Chromatin-associated TMs regulate stress responses and developmental processes related to cold-climate adjustments in *Brachypodium distachyon*, therefore tailoring plant responses to environmental change [19]. There are many factors that affect cold stress tolerance, such as genomic variation, regulation at the transcriptional level, protein translation, epigenetic regulation, and metabolite accumulation [20,21,22,23,24,25]. Wheat under drought, cold, and salt stress can enhance tolerance through epigenetic regulation [26]. Epigenetic regulatory factors recognize heat response processes in various plants [27]. Therefore, epigenetic regulatory factors play an important role under extreme temperatures (Figure 1).

**Figure 2:**
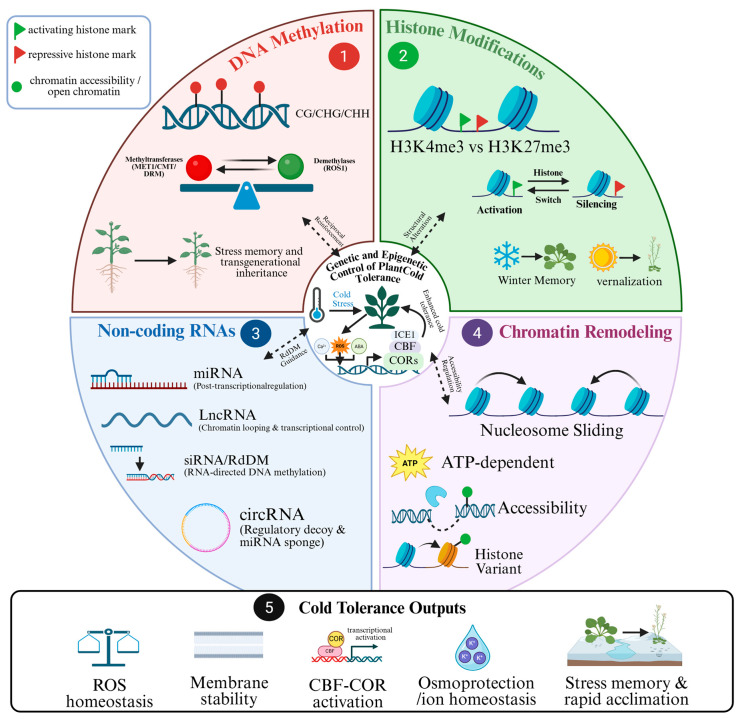
Overview of the epigenetic regulatory network in plant cold stress responses. The diagram depicts a central cold signaling cascade (Ca^2+^–ROS–ABA–*ICE1*–*CBF*–*CORs*) that integrates four core epigenetic modules and their synergistic outputs. (1) DNA Methylation: dynamic balance between methyltransferases (*MET1*, *CMT*, *DRM*) and demethylases (*ROS1*), establishing CG/CHG/CHH methylation patterns for stress memory and transgenerational inheritance. (2) Histone Modifications: the bivalent histone switch (H3K4me3 activation vs. H3K27me3 silencing) mediates winter memory and vernalization. (3) Non-coding RNAs: miRNA (post-transcriptional regulation), lncRNA (chromatin looping), siRNA/RdDM (RNA-directed DNA methylation), and circRNA (regulatory decoy) collectively guide epigenetic silencing. (4) Chromatin Remodeling: ATP-dependent nucleosome sliding, SWI/SNF remodelers, and H2A.Z histone variant deposition enhance chromatin accessibility for cold-responsive genes. (5) Cold Tolerance Outputs: the integrated network culminates in *CBF*–*COR* activation, membrane stability, ROS homeostasis, osmoprotection/ion homeostasis, and stress memory with rapid acclimation. Bidirectional dashed arrows indicate reciprocal crosstalk among modules without implying linear causation. Abbreviations: ABA, abscisic acid; ATP, adenosine triphosphate; *CBF*, C-repeat binding factor; CG/CHG/CHH, cytosine methylation sequence contexts; *COR*, cold-regulated; H3K4me3, histone H3 lysine 4 trimethylation; H3K27me3, histone H3 lysine 27 trimethylation; lncRNA, long non-coding RNA; miRNA, microRNA; RdDM, RNA-directed DNA methylation; ROS, reactive oxygen species; siRNA, small interfering RNA.

Epigenetics pertains to the regulatory mechanisms of gene expression arising from modifications that do not involve changes to the DNA sequence, with effects that can be inherited through cellular division or across generations. Core types of epigenetic modifications include DNA methylation, non-coding RNA regulation, histone modifications, and chromatin remodeling [28,29,30]. Unlike traditional genetic regulation, epigenetic modifications are dynamically reversible, enabling plants to rapidly adapt to environmental changes while maintaining genomic stability, thereby enhancing environmental fitness. DNA methylation represents a highly stable and thoroughly investigated epigenetic change, primarily mediated by DNA methyltransferases that add methyl groups to cytosine, forming 5-methylcytosine. Types of methylation include CG, CHG, and CHH contexts, among which CG methylation is the most conserved in plants [31,32,33]. Here, C stands for cytosine, G stands for guanine, and H stands for any base except guanine. Histone modifications alter histone-DNA binding by covalent modifications such as methylation, acetylation, and ubiquitination, thereby influencing chromatin structure and gene expression. For example, it is well known that gene activation is linked to trimethylation of lysine 4 in histone H3, whereas trimethylation of lysine 27 in H3 is more commonly associated with gene silencing [34,35]. Non-coding RNAs (ncRNAs), including lncRNAs and microRNAs, do not encode proteins but can bind to target genes or chromatin complexes through base complementarity pairing, thereby participating in gene regulation at both transcriptional and post-transcriptional levels [36]. Chromatin remodeling complexes regulate the position and structure of nucleosomes, altering chromatin accessibility to facilitate transcription factor binding. These epigenetic regulatory mechanisms do not exist in isolation but rather interact synergistically to form complex regulatory networks that collectively modulate plant responses to cold stress [37,38,39]. Epigenetics has great potential for the development of plants characterized by enhanced production, superior quality, and improved resistance. DNA methylation, histone modifications, non-coding RNAs, and chromatin remodeling are the four main modules that work together dynamically under cold stress. Below is a sequential description of each module’s molecular mechanisms (Figure 2).

## 2. Precise Regulation of Plant Cold Tolerance by the Dynamic Balance of DNA Methyltransferases and Demethylases

DNA methylation is primarily maintained through the dynamic equilibrium between methyltransferases and demethylases. In plants, the types of genes responsible for GC methylation are DNA methyltransferase 1 (*MET1*); those responsible for CHG and CHH (H = A, C, or T) sites are chromatin methyltransferase (*CMT2*, *CMT3*) and domain-rearrangement methyltransferase (*DRM1*, and *DRM2*) [40,41,42]. Demethylases, on the other hand, comprise the *ROS1* family of proteins, which remove methyl groups via the base excision repair pathway to activate genes [43].

Under cold stress, plants regulate gene expression by modifying the methylation status of key genes through modulation of DNA methyltransferase and demethylase expression [44]. Studies have found that methylation also plays a role in rice’s heat response [45]. Substantial advancements have been achieved in the investigation of epigenetic pathways underlying cold stress in crops such as rice and *citrus*. In rice, environmentally induced DNA methylation variations have been shown to mediate the transgenerational inheritance of cold tolerance traits, providing direct molecular evidence for acquired inheritance. Meanwhile, Liu’s team revealed the role of the *MET1*-*TET8* regulatory module in *citrus* cold resistance, offering a target for cold-resistant breeding in fruit trees [46,47]. Under normal conditions, cold stress induces demethylation in the promoter regions of certain cold-responsive genes, thereby relieving gene expression inhibition and augmenting cold resistance. Simultaneously, it may further optimize the cold resistance regulatory network by silencing negative regulators via methylation [39,44,46]. The dynamic adjustment is species- and tissue-specific, and different plants exhibit distinct response patterns to low-temperature methylation. *Arabidopsis thaliana*, a model species for investigating plant epigenetics, has partially elucidated the DNA methylation mechanisms underlying its cold stress response [48,49]. The study found that cold stress can reduce overall genomic methylation levels in *Arabidopsis*, with the most significant changes observed in the CHH context. These changes are closely associated with alterations in the expression of *DRM* methyltransferases and *ROS1* demethylases [50]. Upregulation of *ROS1* expression under cold stress mediates demethylation of the *COR* gene promoter region, promotes expression of *COR* genes, and enhances plant cold resistance [51,52]. Furthermore, the RNA-guided DNA methylation pathway in *Arabidopsis* is also involved in cold stress response. *DRM* methyltransferases, which carry out methylation at target sites via small interfering RNAs (siRNAs), are mediated by the RdDM pathway. Under cold stress, this pathway can participate in cold resistance regulation by silencing certain negative regulatory genes [53]. In *Arabidopsis*, DNA methylation changes induced by cold stress can also be transmitted to offspring through sexual reproduction. Studies have found that offspring of *Arabidopsis* plants subjected to cold stress exhibit significantly higher survival rates at low temperatures than those of plants that were not subjected to cold stress. This adaptive phenotype is closely associated with methylation differences in specific genomic regions [54]. The intergenerational transmission mechanism of cold stress memory still requires further investigation, such as how methylation modifications are stably maintained during meiosis and whether there are synergistic effects with other epigenetic modifications, which remain unanswered [51,55,56]. However, compared with crops, the DNA methylation regulatory mechanisms in *Arabidopsis* under cold stress are relatively simple, and the findings cannot be fully applied to perennial plants or crops [47,50].

Breakthroughs have been achieved in the study of DNA methylation-mediated cold stress responses in crops, with rice and *citrus* serving as representative examples. Through multi-generational continuous low-temperature treatment of the cold-sensitive rice variety Kitaake, lines with significantly enhanced cold tolerance and stable genetic inheritance were obtained. Further studies revealed that demethylation occurred in the *Dof1* promoter region in this line in the CG and CHG contexts, resulting in *ACT1* expression no longer being inhibited by low temperatures. This demethylation state may be passed down steadily for a minimum of 5 generations [46]. Low temperature inhibits the expression of DNA methyltransferase *MET1b*, leading to impaired maintenance of methylation at the *ACT1* promoter and the formation of a hypomethylated epigenetic allele [57]. The methylation region in the *ACT1* promoter contains a binding site for the transcription factor *Dof1*, and the methylation status affects the binding capacity of *Dof1* [58,59]. *Dof1* preferentially binds to unmethylated promoter regions, thereby activating *ACT1* expression and conferring cold resistance in rice. Natural variation analysis revealed that the methylation status of the *ACT1* gene exhibits a “high in the south, low in the north” gradient distribution. The varieties in low-latitude tropical rice regions predominantly show high methylation, whereas those in high-latitude cool rice regions are predominantly low-methylated. This indicates that epigenetic variation in *ACT1* is a key factor in rice’s adaptation to cold environments during northward migration [60]. Cold stress memory is a phenomenon in which plants store low-temperature experiences through epigenetic changes that can be transmitted to later growth stages or offspring, thereby enhancing environmental adaptability [61]. DNA methylation, a steady epigenetic modification, performs an essential function in the transgenerational transmission of cold-stress memory. The demethylation state of the rice *ACT1* gene can be stably inherited for more than 5 generations and exhibits dominant inheritance. Even when offspring no longer experience low-temperature stress, they can maintain high cold tolerance. This is a classic example of DNA methylation-mediated transgenerational transmission of cold stress memory [62].

**Figure 3:**
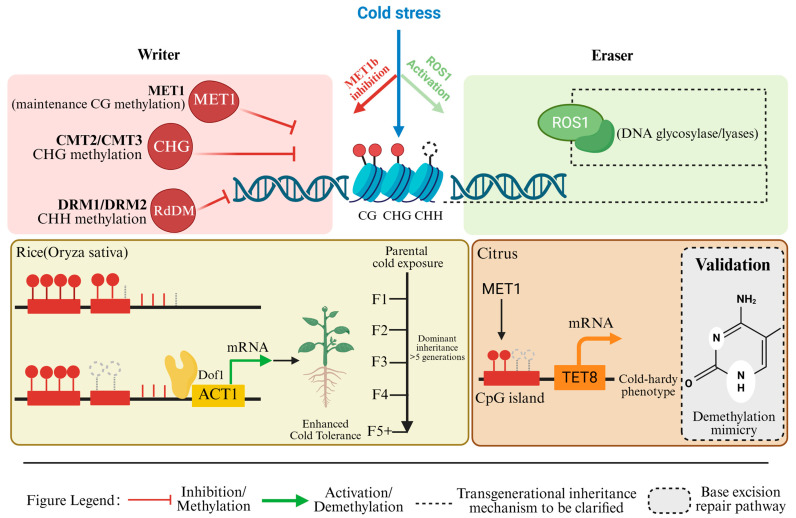
DNA methylation dynamic balance and transgenerational cold memory in plants. The upper panel shows the dynamic balance between DNA methyltransferases (*MET1*, *CMT2*/*3*, *DRM1*/*2*) and demethylases (*ROS1*) under cold stress, with CG/CHG/CHH methylation contexts indicated. The lower left panel shows rice *ACT1* promoter demethylation enabling *Dof1* binding and dominant transgenerational inheritance (F1–F5+). The lower right panel shows *citrus TET8* CpG island demethylation regulated by *MET1*, with 5-azacytosine validation. Red lines represent inhibition/methylation; green lines represent activation/demethylation; filled red circles indicate methylated cytosines, whereas open circles indicate demethylated/unmethylated cytosines; dashed lines indicate transgenerational inheritance; the dashed box indicates the base excision repair pathway.

In *citrus*, the role of the *MET1*-*TET8* regulatory module in cold stress response was elucidated. Through combined methylation and transcriptome analysis of cold-resistant Yichang orange and cold-sensitive lemon, 33 key genes were identified as highly expressed and hypomethylated in Yichang orange, with the most significant changes observed in the expression and methylation of the *TET8* gene [47]. The *TET8* gene is primarily methylated at CpG sites, and its methylation level is regulated by *MET1*. Under cold stress, *MET1* expression in Yichang orange is reduced, leading to decreased methylation of the *TET8* gene and upregulation of its expression, thereby improving the plant’s cold resistance [61]. Treatment of *citrus* with the DNA methylation inhibitor 5-azacytosine reduced *TET8* methylation, increased its expression, and significantly enhanced cold resistance, demonstrating the functionality of this regulatory module and providing a potential target for cold-resistant breeding in *citrus* [63,64,65]. In many plants, MET is the main functional gene in the methylation process. Under cold stress, the equilibrium between DNA demethylases (*ROS1*, via base excision repair) and methyltransferases (*MET1*, *CMT2*/*3*, *DRM1*/*2*) is shifted. In rice, cold-induced inhibition of *MET1b* leads to hypomethylation at the *ACT1* promoter in CG and CHG contexts, enabling *Dof1* transcription factor binding and activation of *ACT1* expression, thereby conferring enhanced cold tolerance and allowing dominant inheritance for over five generations (Figure 3).

## 3. Role of Histone Modifications in Plant Cold Stress Responses

Core components of eukaryotic chromatin, called nucleosomes, are histone octamers, which include two molecules of each histone subtype (H2A, H2B, H3, and H4), and around 147 base pairs (bp) of DNA [66]. Histone modification refers to the covalent modification of amino acid residues at the N-terminal tail of histones by histone-modifying enzymes, including various types such as acetylation, ubiquitination, methylation, and phosphorylation. Similarly, phosphatases, demethylases, deacetylases, and deubiquitinases can remove these reversible epigenetic marks [67]. Different combinations of these modification types form the ‘histone code,’ which regulates gene expression [68].

Histone acetyltransferases are enzymes that catalyze histone acetylation, a process commonly linked to gene activation. It weakens the interface between DNA and histones, loosening chromatin and facilitating the binding of transcription factors. Conversely, histone deacetylation, catalyzed by histone deacetylases, leads to chromatin condensation and inhibits gene expression [69]. In *Arabidopsis*, *GCN5* is a conserved HAT that is upregulated by cold stress, mediating elevated H3 acetylation in the promoter regions of *CBF* and *COR* genes, thereby promoting their expression [70]. The study revealed that the gcn5 mutant *Arabidopsis* exhibited significantly enhanced cold sensitivity, whereas overexpression of *GCN5* improved the plant’s cold resistance, demonstrating the core role of *GCN5* in regulating histone acetylation under cold stress [71]. The dynamic equilibrium of histone acetylation/deacetylation is essential for plants to react to cold stress. In *Arabidopsis thaliana*, *HOS15* is a key histone deacetylase-related protein. Under normal temperature conditions, *HOS15* binds to HD2C and inhibits *COR* gene expression by deacetylation [72,73]. *HOS15* interacts with *CUL4* ubiquitin ligase to promote HD2C ubiquitination and degradation under cold stress, thereby relieving *COR* gene inhibition. Simultaneously, *HOS15* also recruits the *CBF* transcription factor to the *COR* gene promoter, facilitating histone H3 acetylation and elevating the plant’s cold tolerance through boosting *COR* gene expression [74].

The functions of histone methylation are diverse, contingent upon the modification site and degree of methylation. For instance, it is common to associate H3K4me3 and H3K36me3 with the activation of gene transcription, whereas H3K9me2 and H3K27me3 are related to the silencing of gene expression [75]. Under cold stress, plants rapidly activate or silence temperature-related genes by adjusting the activity of histone modification enzymes, thereby altering the histone modification status of cold-responsive genes to adapt to low-temperature environments [74]. The H3K27me3-mediated gene silencing mechanism is the most extensively studied [76,77,78]. In *Arabidopsis*, the polycomb repressive complexes 1 (PRC1) and 2 (PRC2) play roles in the establishment and maintenance of H3K27me3 (trimethylation of lysine 27 on histone H3). PRC2 catalyzes H3K27me3 to initiate gene silencing, while PRC1 maintains the silencing state by catalyzing H2Aub (histone H2A monoubiquitination) [79]. Temperature regulates plant cell fate by modulating the dynamics of PRC1-H2A. Zub and PRC2-H3K27me3. Under low-temperature conditions, PRC2 mutants exhibit increased enrichment of H2A.Z at embryonic key gene loci, which, together with PRC1, suppresses gene expression. This enables the mutants to develop normal plant structures, whereas at normal temperature (22 °C), they undergo callus formation [80]. In addition to H3K27me3, other histone methylation modifications are also involved in the cold stress response. In *Arabidopsis*, cold stress can increase H3K4me3 levels in the promoter region of the *CBF* gene, thereby promoting *CBF* gene expression, while decreasing H3K9me2 levels, thereby releasing gene silencing [81,82]. In rice, upregulation of the *OsTrx1* gene expression under cold stress mediates increased H3K4me3 modification, activates cold-resistant gene expression, and enhances cold tolerance. Conversely, *OsJMJ703* can activate cold-responsive genes by removing the H3K27me3 modification, thereby participating in rice’s adaptation to cold stress [50]. Histone methylation is also involved in the formation and maintenance of cold stress memory. In *Arabidopsis*, He Yuehui’s team discovered that wintering low temperatures induce the *FLC* gene to form an H3K27me3-mediated polycomb silencing domain, leading to *FLC* gene silencing. This silencing state remains stably maintained after spring warming, endowing plants with “wintering memory” and ensuring normal spring flowering [83]. Further studies revealed that the “cold memory element” region of the *FLC* gene exhibits a bivalently chromatinized state, coexisting with H3K4me3 and H3K27me3. Plant-specific EBS and SHL proteins can form dimers, recognize this bivalent modification, recruit the VIN3-PRC2 complex, and promote the enrichment of H3K27me3 in the CME region. Upon warming, PRC2 diffuses to the entire *FLC* locus, maintaining H3K27me3 and establishing a stable silencing state, thereby sustaining winter memory [84].

Histone ubiquitination exhibits synergistic effects with modifications such as methylation and acetylation in regulating plant cold stress responses. In *Arabidopsis*, H2Aub, catalyzed by PRC1, and H3K27me3, catalyzed by PRC2, work synergistically to maintain the cold stress-induced gene silencing state [85]. PRC1 inhibits the expression of embryonic development genes by catalyzing the mono-ubiquitination of histone variant H2A.Z, while PRC2 maintains this inhibitory state through H3K27me3. The two proteins act synergistically to regulate plant cell-fate determination under cold stress. Additionally, H2Bub participates in the cold stress response. In *Arabidopsis*, H2Bub modification promotes the establishment of H3K4me3, thereby activating *CBF* pathway gene expression and enhancing plant cold resistance [86]. Histone ubiquitination regulates gene expression by modifying chromatin structure or recruiting other regulatory factors. For instance, monoubiquitination of histone H2A is commonly linked to gene silencing, whereas monoubiquitination of H2B promotes gene transcription [87].

Phosphorylation of histones mainly occurs on the N-terminal domains of serine and threonine [66]. Multiple serine and threonine sites on H2A and H3 in plants are phosphorylated and dynamically modified during chromosome division [66,88]. Phosphorylation of H3 often occurs in the centromeric region and the pericentromeric region [89,90,91]. Phosphorylation of histones may be involved in chromosome condensation and disappears along with chromosome decondensation [92]. During metaphase and anaphase, Haspin-mediated histone H3 threonine 3 phosphorylation occurs at centromeres, whereas during meiosis it occurs at telomeres. Chromosome misalignment and segregation were observed during mitosis in *ZmHaspin* mutants, which were associated with reduced H3T3 and histone H3 serine 10 phosphorylation [93]. Phosphorylation plays an important role in the cell cycle, and dephosphorylation is equally important. The application of specific inhibitors of PP2A and PP1 phosphatases (cantharidin) has been detected to result in high levels of phH3Ser10 along the chromosome arms during mitosis [94]. In BY-2 cells subjected to high salt and low temperature stress, changes in the phosphorylation of histone H3 are associated with the activation of stress-responsive genes [95]. There is relatively little research on histone phosphorylation under temperature stress, and further in-depth study is needed.

There is also a synergistic regulatory relationship between DNA methylation and histone modifications. In the regulation of the rice *ACT1* gene, DNA demethylation promotes *Dof1* binding, and *Dof1* may further recruit histone modification enzymes to alter histone modification status at the *ACT1* gene, thereby enhancing gene expression [46]. In *citrus*, the DNA methylation status of the *TET8* gene correlates with histone acetylation levels. Under low-methylation conditions, elevated histone acetylation at the *TET8* gene promoter region enhances gene expression. This synergistic interaction among different epigenetic modifications forms a sophisticated regulatory network, ensuring rapid and accurate plant responses to cold stress [96].

## 4. The Regulatory Network of Non-Coding RNA in Plant Cold Stress Response

miRNA is a class of non-coding single-stranded RNA with a length of 20–24 nucleotides (nt). It mediates the degradation or translation inhibition of target mRNA by complementary base pairing, thereby regulating gene expression at the post-transcriptional level. Non-coding RNAs are involved in plant growth, yield, and responses to biotic and abiotic stresses [97,98,99,100,101,102]. For example, miRNAs, a type of non-coding RNA, play a crucial role in plant responses to cold stress by modifying key genes in cold-responsive pathways, thereby influencing plant cold resistance [61]. Research on miRNAs and cold stress has been reported across various plants [101,103,104,105,106]. A research team discovered that *miR528* influences the balance of reactive oxygen species in banana peels by regulating the expression of polyphenol oxidase, thereby inducing cold-induced browning [107]. Under cold stress, *miR156c* is significantly upregulated in banana peel, whereas its target gene, *MaSPL4*, is downregulated. The promoter of *MIR528* is regulated by *MaSPL4*, which enhances its expression. Consequently, downregulation of *MaSPL4* inhibits *miR528* accumulation, leading to upregulation of the *PPO* gene and a significant increase in reactive oxygen species. Additionally, *MaSPL4* affects the synthesis of long-chain fatty acids and cutin in the peel, disrupting their structural stability and ultimately leading to cold-damage phenotypes [108]. This *miR156c*-*MaSPL4*-*miR528*-*MaPPO* model pathway reveals a cold-stress response independent of the *CBF* classical pathway, thereby enriching the plant cold-stress regulation network. In *Arabidopsis*, *miR169* contributes to the cold stress response [109]. On the other hand, *miR397* influences lignin synthesis by targeting the laccase gene, thereby improving *Arabidopsis* cold tolerance [110]. Knockdown of *MIR1868* increased pollen fertility, seed setting, and grain yield under cold stress in rice, whereas its overexpression conferred the opposite result [111]. These studies indicate that miRNAs establish a complex regulatory network in response to cold stress by targeting genes with diverse functions, thereby playing multifaceted roles in plant cold resistance [112].

lncRNA refers to non-coding RNA with a length exceeding 200 nucleotides. Although it does not encode proteins, lncRNA can participate in gene regulation through various mechanisms, such as serving as molecular scaffolds, guides, or decoys, thereby influencing chromatin structure, transcription factor activity, or RNA stability [113]. The epigenetic regulatory network of the *CBF* pathway has been elucidated in *A. thaliana*, including histone modifications that activate *CBF* gene expression and lncRNA-mediated fine-tuning of *CBF* expression [50]. In recent years, the function of lncRNAs in the response of plants to cold stress has attracted attention, with their regulatory mechanisms primarily involving cis- and trans-regulation. In *Arabidopsis*, the lncRNAs *SVALNA* and *SVALKA* participate in the cold stress response by regulating the expression of *CBF1* and *CBF3*. Studies have revealed that SVK possesses both cis-and trans-regulatory functions, modulating the expression of *CBF1* and *CBF3* through RNA polymerase II and chromatin remodeling mechanisms. In contrast, SVN exerts only cis-regulatory effects, negatively regulating *CBF3* expression via the RNA polymerase II collision mechanism [114]. Furthermore, SVK exhibits multiple alternative splicing isoforms, the stability of which may be closely related to regulatory functions, indicating that the structural diversity of lncRNAs significantly influences their biological roles. Another lncRNA, *COOLAIR*, is involved in *Arabidopsis* vernalization by regulating *FLC* gene expression [115]. *COOLAIR* is an antisense lncRNA upstream of the *FLC* gene. Under cold stress, *COOLAIR* expression is upregulated, recruiting the PRC2 complex to the *FLC* gene locus, promoting H3K27me3 modification and suppressing *FLC* gene expression, thereby assisting plants in forming winter memory [116,117]. The *COOLAIR* expression is regulated by histone modifications and DNA methylation, forming a complex epigenetic regulatory circuit that ensures the stable silencing of *FLC* genes under low temperatures.

Plant responses to cold stress involve not just miRNAs and lncRNAs, but also small interfering RNAs (siRNAs). siRNAs can modulate gene expression through mediating DNA methylation via the RdDM pathway, silencing target genes, or directly degrading target mRNA [31,118]. In *A. thaliana*, cold stress can induce the expression of specific siRNAs that silence genes negatively regulated by cold resistance via the RdDM pathway, thereby enhancing plant cold tolerance. In rice, siRNAs regulate *ACT1* expression by modulating DNA methylation, thereby influencing cold resistance [119].

Circular RNAs (circRNAs) are novel types of non-coding RNAs that show promise in plants’ reactions to cold stress. CircRNAs can regulate target genes via miRNA adsorption, thereby forming circRNA-epigenetic networks [120]. In wheat, multiple circRNAs exhibit substantial changes in expression levels under cold stress, regulating miRNA and target gene expression through the ceRNA mechanism, thereby participating in cold stress adaptation. Further investigation into the precise regulatory mechanisms and activities of circular RNAs is necessary, as the field of research on their role in cold stress in plants is still in its early stages [121,122,123,124].

**Figure 4:**
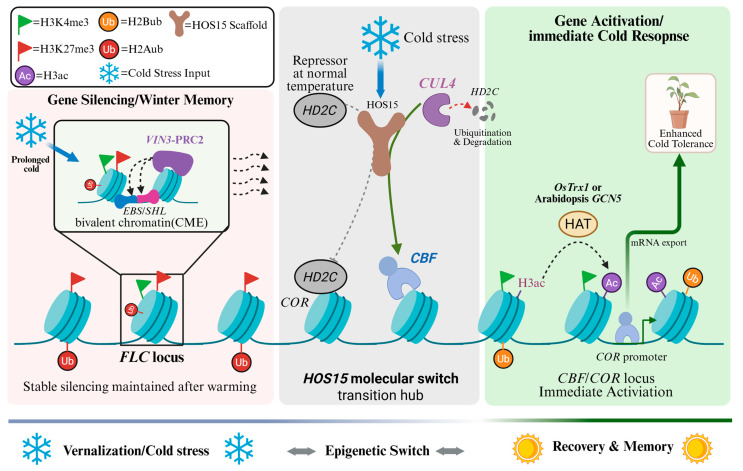
The histone code for cold-responsive gene activation and silencing in plants. The left panel depicts the *FLC* locus with H3K27me3-mediated silencing, the CME bivalent chromatin state (H3K4me3 and H3K27me3), and EBS/SHL-mediated VIN3-PRC2 recruitment for winter memory. The central panel shows the *HOS15* switch recruiting *CUL4* to degrade *HD2C* and recruiting *CBF* to activate *COR*. The right panel shows the *CBF*/*COR* locus with activating marks (H3K4me3, H3ac, H2Bub). The legend indicates: green flag, H3K4me3; red flag, H3K27me3; purple Ac, H3ac; orange Ub, H2Bub; red Ub, H2Aub; snowflake, cold stress input; Y-shape, *HOS15* scaffold.

During vernalization, the *FLC* locus is stably silenced by H3K27me3and H2Aub, establishing winter memory. The CME region exhibits a bivalent chromatin state with coexisting H3K4me3 and H3K27me3. The EBS/SHL dimer recognizes this bivalent modification, recruits the VIN3-PRC2 complex to promote H3K27me3 enrichment, and, upon warming, PRC2 diffuses across the entire *FLC* locus to maintain stable silencing. Note that *FLC* silencing requires prolonged cold/vernalization. *HOS15* functions as an epigenetic switch: at normal temperature, it binds HD2C to repress *COR* genes via deacetylation; under cold stress, it recruits the *CUL4* ubiquitin ligase to promote HD2C ubiquitination and degradation, while simultaneously recruiting the *CBF* transcription factor to facilitate H3 acetylation at the *COR* promoter. At the *CBF*/*COR* locus, activating marks including H3K4me3, H3ac, and H2Bub promote chromatin opening and immediate transcription of cold-responsive genes, conferring enhanced cold tolerance. *CBF*/COR activation triggers an immediate cold response, in addition to the prolonged vernalization process at *FLC* (Figure 4).

## 5. The Mechanism of Chromatin Remodeling and Cold Stress Response

Chromatin remodeling is a process that regulates gene expression by adjusting the position, structure, or composition of nucleosomes through chromatin remodeling complexes, thereby altering chromatin accessibility. These complexes primarily include the SWI/SNF, ISWI, and INO80 families, which provide energy via ATP hydrolysis to move nucleosomes or replace histone variants, enabling transcription factors to bind to DNA and repress or activate gene expression [72,125,126,127]. The replacement of histone variants is a key form of chromatin remodeling, with distinct histone variants performing distinct functions. The histone variant H2A.Z plays a critical role in plant temperature sensing by altering chromatin structure, thereby influencing the expression of temperature-responsive genes [119]. Under cold stress, plants regulate gene expression by adjusting nucleosome positioning and histone variant composition through chromatin remodeling complexes, thereby altering the chromatin accessibility of cold-responsive genes to achieve adaptation to low-temperature environments [120]. Studies in *Arabidopsis* have revealed mechanisms of chromatin remodeling and histone modification that synergistically regulate the cold stress response. The transcription factor TOE1 interacts with the INO80 chromatin remodeling complex to remove H2A.Z from the nucleosomes of key genes [128,129], and low temperatures attenuated this process by suppressing TOE1 expression, resulting in the accumulation of PRC1-H2A. Zub on embryonic key genes, which inhibited gene expression and altered cell fate [130]. This mechanism demonstrates that chromatin remodeling, through regulation of histone variant distribution, synergizes with histone modifications to modulate plant adaptations to low temperature jointly [87]. One of the factors that *Arabidopsis* uses to respond to cold stress is the chromatin remodeling factor *PKL* [131]. The chromatin state of cold-responsive genes, including *COR*, is regulated by *PKL*, a chromatin remodeling protein belonging to the CHD3 family, through processes that rely on H3K27me3 [132]. Under normal temperature conditions, *PKL* inhibits *COR* gene expression by promoting H3K27me3 modification. Under cold stress, *PKL* function is suppressed, causing a decline in H3K27me3 levels at the *COR* gene, resulting in gene activation and enhanced cold resistance in the plant [76,133,134,135]. Furthermore, *PKL* interacts with histone deacetylases to co-regulate the expression of cold-responsive genes, further elucidating the synergistic relationship between chromatin remodeling and other epigenetic modifications [136].

Chromatin remodeling exhibits close synergy with DNA methylation and histone modifications in jointly regulating the plant cold stress response [137]. In rice, after DNA demethylation in the promoter region of the *ACT1* gene, the chromatin remodeling complex may be recruited to this region, adjusting nucleosome positioning to facilitate binding of the *Dof1* transcription factor, thereby activating gene expression [46]. In the regulation of *Arabidopsis FLC* genes, the chromatin remodeling complex participates in the replacement of H2A.Z, while the presence of H2A.Z facilitates the recognition of bivalent chromatin marks by EBS/SHL proteins, promoting the establishment of H3K27me3 modifications and forming a stable silencing state [138,139,140,141,142]. Furthermore, chromatin remodeling may potentially affect the expression of non-coding RNAs. Studies have revealed that chromatin remodeling complexes can modulate the accessibility of lncRNA genes, thereby regulating lncRNA transcription [143]; lncRNAs can further influence chromatin structure by recruiting chromatin remodeling complexes to target gene loci, thereby forming regulatory loops [144]. This multi-level synergy ensures the stability and precision of the plant’s cold stress response regulatory network.

## 6. Species-Specific and Synergistic Networks of Epigenetic Regulation of Plant Cold Stress

### 6.1. Epigenetic Regulation Differences Among Different Plant Groups

The epigenetic regulation of plants under cold stress exhibits significant species specificity, which is closely related to their evolutionary history, growth environment, and cold resistance [145]. *Arabidopsis thaliana*, as a temperate herbaceous plant, exhibits strong cold acclimatization capacity. Its epigenetic regulation of cold stress is centered on the *CBF* pathway, forming a complex network through histone modifications and lncRNA regulation. Moreover, cold stress memory is primarily maintained via H3K27me3 modification [146]. Rice, a crop native to tropical regions, primarily relies on DNA methylation for epigenetic regulation under cold stress. Methylation variation in the *ACT1* gene is crucial for its adaptation to low-temperature environments in high latitudes, and this epigenetic variation can be transmitted across generations [46]. As a perennial woody plant, *citrus* species rely on the *MET1*-*TET8* regulatory module to respond to cold stress, which modulates key gene expression through DNA methylation. The perennial nature of woody plants may endow them with a more complex cold-stress memory mechanism [147]. These species differences indicate that plants have evolved epigenetic regulatory strategies adapted to their growth environments. An in-depth investigation of regulatory mechanisms across different species contributes to a comprehensive understanding of the evolutionary patterns of cold adaptation in plants [148].

### 6.2. Synergistic Interactions Among Different Epigenetic Regulatory Pathways

The various epigenetic regulatory pathways involved in plant cold stress responses are not isolated but rather interact synergistically to form a complex regulatory network [149]. DNA methylation can influence chromatin structure by providing binding sites for histone-modifying enzymes and chromatin remodeling complexes; histone modifications, in turn, recruit non-coding RNAs to regulate gene expression [150,151,152,153]. Non-coding RNAs can, in turn, influence DNA methylation and histone modification states, thereby forming regulatory loops [154]. Taking the *Arabidopsis FLC* gene as an example, under cold stress, *COOLAIR* lncRNA expression is upregulated, recruiting the PRC2 complex to the *FLC* locus and promoting H3K27me3 modification [117,155,156]. Meanwhile, the EBS/SHL protein recognizes bivalent chromatin markers, further enhancing the H3K27me3 modification; the chromatin remodeling complex then adjusts nucleosome positioning to facilitate the diffusion of the PRC2 complex [157]. Furthermore, the DNA methylation status of *FLC* loci also influences these processes, collectively maintaining the silencing of *FLC* genes and forming winter memory [158]. In rice, DNA demethylation of the *ACT1* gene promotes *Dof1* binding, which may recruit histone acetyltransferases to increase histone acetylation levels. Concurrently, the chromatin remodeling complex adjusts nucleosome positioning, further activating gene expression and enhancing cold resistance [46,159,160]. This multi-pathway, coordinated regulatory network allows plants to swiftly and accurately respond to cold stress while preserving genomic stability and plasticity [161].

## 7. Research Prospects and Application Perspectives

### 7.1. Problems and Bottlenecks in Current Research

Despite significant progress in understanding epigenetic regulatory mechanisms under plant cold stress, numerous scientific questions and research bottlenecks remain. The mechanisms underlying the synergistic interactions among different epigenetic modifications have not been fully elucidated, and the interaction networks of various regulatory factors remain to be further analyzed. In particular, the regulatory relationships among non-coding RNAs, DNA methylation, and histone modifications remain unclear, with most studies still limited to correlation analysis rather than establishing causal relationships [162]. The mechanism underlying the intergenerational transmission of cold stress memory remains unclear. How methylation and histone modifications are stably maintained during meiosis, and whether specific regulatory factors exist to ensure memory transmission, remain unanswered and require further in-depth research [163].

Furthermore, research on the epigenetic mechanisms underlying cold stress responses in perennial and woody plants remains relatively underdeveloped. Current studies predominantly focus on annual plants such as *Arabidopsis* thaliana and rice, while the long-term cold-stress adaptation and memory mechanisms in perennial plants may differ from those in annual species [164]. Meanwhile, the dynamic detection technology for epigenetic modifications still requires optimization. How to monitor epigenetic modifications in plants under cold stress in real time, as well as the dynamic correlations between these changes and gene expression and phenotypes, remains a technical bottleneck in current research [165]. Finally, the application of epigenetic editing technology in cold-resistant crop breeding is still in its infancy. The key challenge lies in efficiently and precisely editing epigenetic loci while avoiding off-target effects, which is crucial for its practical application in agricultural production [166].

### 7.2. Future Research Direction

To address the existing issues in current research, future studies could focus on the following aspects: First, employing multi-omics technologies to analyze the synergistic interaction networks among different epigenetic modifications systematically, elucidate the interaction relationships and molecular mechanisms of various regulatory factors, and construct a comprehensive epigenetic regulatory network model for cold stress [167]. Secondly, the focus is on the intergenerational transmission mechanism of cold-stress memory, with in-depth research into the maintenance mechanisms of epigenetic modifications during meiosis, the identification of key regulatory factors involved in memory transmission, and the provision of more comprehensive molecular evidence to better understand acquired inheritance [55,168,169,170].

Thirdly, it is necessary to strengthen research on the epigenetic regulation of cold stress in perennial and woody plants, to compare regulatory mechanisms across plant life forms, and to reveal the evolutionary laws of plant cold adaptation [148]. Fourth, develop and optimize epigenetic dynamic detection technologies, such as real-time fluorescence quantitative detection and single-cell epigenomics, to enable real-time monitoring of epigenetic modification changes under cold stress and further elucidate the causal relationship between epigenetic modifications and phenotypes [171,172,173,174]. Fifth, explore cross-interactions between epigenetic regulation and other signaling pathways to comprehensively understand the molecular processes governing plant responses to cold stress [175,176].

### 7.3. Application Prospects of Cold-Resistant Breeding

The study of epigenetic regulatory mechanisms provides novel insights and strategies for cold-resistant crop breeding, with broad application prospects. Firstly, by identifying key epigenetic loci responsive to cold stress, epigenetic molecular markers can be developed for screening and identifying cold-resistant germplasm resources, thereby enhancing breeding efficiency [177]. Secondly, epigenetic editing technology is used to target epigenetic loci in crops, thereby regulating the expression of cold-resistant genes and breeding crop varieties with enhanced cold resistance [178,179,180].

For instance, targeted reduction in methylation levels in the promoter region of the rice *ACT1* via the CRISPR/dCas9 system can enhance *ACT1* gene expression and improve cold tolerance in rice [46]. In *citrus*, editing the *MET1* gene to reduce *TET8* methylation can enhance cold resistance [47]. Furthermore, epigenetic regulatory factors can be used to develop plant cold-resistance modulators, thereby enhancing crop cold tolerance through exogenous treatment and mitigating the impact of sudden low-temperature disasters [181].

However, epigenetic breeding still faces numerous challenges, such as the stability of epigenetic modifications and the efficiency and specificity of editing technologies, both of which require further optimization and refinement. With the ongoing advancement of technology, research on epigenetic regulatory mechanisms will yield more effective strategies for cold-resistant crop breeding, thereby making significant contributions to global food security [182].

## 8. Conclusions

The epigenetic regulation of plant responses to cold stress constitutes a complex network system. Through coordinated mechanisms including histone modifications, DNA methylation, non-coding RNA regulation, and chromatin remodeling, this system dynamically adjusts gene expression patterns to enable plants to rapidly adapt to low-temperature environments and establish stable stress memory [183]. DNA methylation regulates gene expression and mediates the transgenerational transmission of cold tolerance traits by altering the methylation status of key genes [46]. Histone modifications finely regulate the activation and silencing of cold-responsive genes by forming a ‘histone code’ and participate in the formation and maintenance of stress memory [39,76,184]. Non-coding RNAs establish diverse regulatory pathways through mechanisms such as miRNA-mediated post-transcriptional regulation and lncRNA-mediated chromatin regulation [185]. Chromatin remodeling alters gene chromatin accessibility by modifying nucleosome structure and histone variant composition, thereby enabling other epigenetic modifications [186].

The epigenetic regulation of cold stress across different plant groups exhibits significant species specificity, reflecting the adaptive evolution of plants to their growth environments [75]. Although current studies have revealed some regulatory mechanisms, issues such as unclear mechanisms of synergistic interactions, ambiguous mechanisms of cross-generational transmission, and insufficient research on perennial plants remain unresolved [169,187,188]. In the future, through the application of multi-omics technologies and epigenetic editing techniques, in-depth analysis of epigenetic regulatory networks is expected to elucidate further the molecular mechanisms underlying plant cold adaptation [189]. Meanwhile, the crop cold resistance breeding strategy based on epigenetic mechanisms will provide new solutions for breeding cold-resistant crop varieties and for coping with low-temperature disasters caused by global climate change, which has important theoretical value and application prospects [190].

In conclusion, the epigenetic regulation of plant responses to cold stress constitutes a complex network of multi-pathway synergy, with DNA methylation, non-coding RNA regulation, and histone modifications forming the core regulatory modules (Table 1) [187]. DNA methylation mediates the transgenerational inheritance of cold tolerance traits through dynamic reprogramming, providing molecular evidence for acquired inheritance. lncRNAs and antisense RNAs can target the regulation of *CBF* family genes via chromatin remodeling, R-loop formation, and other mechanisms, thereby modulating the cold acclimation process [114,191]. The antagonistic distribution of H3K4me3 and H3K27me3 in histone modifications challenges the traditional understanding of single-modification switches, demonstrating the precision of regulatory mechanisms. Current research has identified key regulatory sites and pathways, but the interaction networks among different mechanisms and the specific regulation of cold damage in horticultural crops remain to be elucidated [192,193,194]. The integration of epigenetic editing technologies in the future, coupled with in-depth comparative studies of cross-species mechanisms, could establish a novel paradigm for molecular breeding of cold-resistant crops, thereby addressing agricultural challenges posed by global climate warming. Precise breeding at the molecular level greatly reduces time spent selecting high-quality germplasm. Breeding designs based on multiple dynamic epigenetics are more in line with future breeding trends.

## Figures and Tables

**Table 1:** Summary of key epigenetic regulators and their functions under cold stress in plants.

Gene/Protein	Species	Epigenetic Category	Expression/Activity Change Under Cold Stress	Molecular Mechanism	Biological Function	Reference
*MET1/MET1b*	*Oryza sativa* L., *Citrus*	DNA methylation (maintenance)	Expression inhibited/reduced	Impaired maintenance of CG methylation at target promoters; hypomethylation enables transcription factor access	Negative regulator (relief of repression)	[40,41,42,46,47,57]
*ROS1*	*Arabidopsis*	DNA demethylation	Expression upregulated	Removes methyl groups via base excision repair; demethylates *COR* gene promoters to activate expression	Positive regulator of cold tolerance	[43,50,51,52]
*ACT1*	*Oryza sativa* L.	Target gene (epigenetically regulated)	Promoter hypomethylation (CG/CHG)	Loss of methylation allows Dof1 binding and transcriptional activation; epigenetic allele stably inherited for >5 generations	Dominant transgenerational cold tolerance	[46,57,58,59,60]
*TET8*	*Citrus*	Target gene (epigenetically regulated)	Promoter CpG demethylation	MET1 reduction leads to decreased methylation and elevated *TET8* expression	Enhanced cold resistance in *citrus*	[47,61]
*GCN5*	*Arabidopsis*	Histone acetyltransferase (HAT)	Expression upregulated	Catalyzes H3 acetylation at *CBF* and *COR* promoters; promotes chromatin opening	Activation of cold-responsive transcription	[70,71]
*HOS15*	*Arabidopsis*	Scaffold/switch protein	Activity switched (not expression)	Recruits *CUL4* ubiquitin ligase to degrade HD2C; simultaneously recruits *CBF* to *COR* promoters	Epigenetic switch from repression to activation	[72,73,74]
HD2C	*Arabidopsis*	Histone deacetylase (HDAC)	Ubiquitinated and degraded	Repression of *COR* is relieved upon HD2C removal	Negative regulator (removed under stress)	[72,73]
*FLC*	*Arabidopsis*	Target gene (memory)	Silenced by H3K27me3 spreading	CME bivalent state recognized by EBS/SHL → recruits VIN3-PRC2; PRC2 diffuses across locus upon warming	Winter memory; vernalization ensuring spring flowering	[83,115]
*OsTrx1*	*Oryza sativa* L.	H3K4 methyltransferase	Expression upregulated	Increases H3K4me3 at cold-responsive gene promoters	Activation of cold-tolerance genes	[50]
*OsJMJ703*	*Oryza sativa* L.	H3K27me3 demethylase	Active (implied)	Removes H3K27me3 repressive marks from cold-responsive genes	Activation of cold-responsive genes	[50]
EBS/SHL	*Arabidopsis*	Bivalent histone reader	Active (implied)	Forms dimers to recognize H3K4me3/H3K27me3 bivalent marks; recruits VIN3-PRC2 to *FLC*	Establishment of winter memory	[84]
VIN3	*Arabidopsis*	PRC2-associated protein	Cold-induced	Associates with PRC2 to catalyze H3K27me3 at the *FLC* locus	Vernalization-mediated epigenetic silencing	[84]
*miR1432*	*Oryza sativa* L.	miRNA	Expression regulated	Targets OsACAs (Ca^2+^-ATPase) to impair Ca^2+^ homeostasis and negatively regulate cold tolerance	Negative regulator of cold tolerance	[103]
NAT1850 (pri-miR1850)	*Oryza sativa* L.	NAT/lncRNA	Expression upregulated	Cis-natural antisense transcript of pri-miR1850; represses NPR3 to negatively regulate cold tolerance	Negative regulator of cold tolerance	[106]
*miR156c*	*Musa acuminata* ‘(AAA)’	miRNA	Significantly upregulated	Suppresses *MaSPL4* → inhibits *miR528* → *MaPPO* upregulation → ROS burst	Negative regulator of cold tolerance (tropical sensitive crop)	[108,109]
*miR169*	*Arabidopsis*	miRNA	Expression downregulated	Derepression of *NF-YA* transcription factor → activation of *COR* genes	Positive regulator of cold tolerance	[109]
*miR172*	*Oryza sativa* L.	miRNA	Expression regulated	Regulates *AP2/ERF* transcription factor; overexpression enhances rice cold tolerance	Positive regulator of cold tolerance	[112]
*miR397*	*Arabidopsis*	miRNA	Expression regulated	Targets laccase gene to influence lignin synthesis	Improves cold tolerance	[110]
*miR528*	*Musa acuminata* ‘(AAA)’	miRNA	Accumulation inhibited	Targeted by *MaSPL4;* reduced accumulation leads to PPO upregulation and ROS burst	ROS homeostasis regulator; negative regulator of cold tolerance in tropical crops	[107,108]
*COOLAIR*	*Arabidopsis*	lncRNA	Expression upregulated	Recruits PRC2 complex to *FLC* locus; promotes H3K27me3 establishment	Assists winter memory formation	[116,117]
*SVALKA/SVALNA*	*Arabidopsis*	lncRNA	Cold-induced (expression)	*SVALKA*: cis- and trans-regulation of *CBF1*/*CBF3* via RNA Pol II collision; *SVALNA*: cis-regulation of *CBF3*	Fine-tuning of *CBF* cold signaling	[114]
H2A.Z	*Arabidopsis*	Histone variant	Removal attenuated under cold	TOE1-INO80 removes H2A.Z from nucleosomes; cold suppresses TOE1 leading to accumulation	Temperature sensing; cell fate determination	[80,128,129]
*PKL*	*Arabidopsis*	Chromatin remodeler (CHD3 family)	Function suppressed	Decreases H3K27me3 at *COR* locus; interacts with HDACs to co-regulate cold-responsive genes	Relief of repression; activation of *COR*	[131,132,136]
TOE1	*Arabidopsis*	Transcription factor (interacts with INO80)	Expression suppressed by low temperature	Interacts with INO80 complex to remove H2A.Z from nucleosomes; attenuated under cold stress	Temperature sensing via H2A.Z dynamics	[128,129,130]
*MaSPL4*	*Musa acuminata* ‘(AAA)’	Transcription factor (*miR156c* target)	Expression downregulated	Target of *miR156c;* normally binds to *MIR528* promoter and activates cutin synthesis in peel	Mediator of *miR156c*-dependent cold damage phenotype	[108]

## Data Availability

No new data were created or analyzed in this study. Data sharing is not applicable to this article.

## Abbreviations

The following abbreviations are used in this manuscript: *ACT1*: actin 1; AP2/ERF: APETALA2/ethylene-responsive factor; BY-2: Bright Yellow 2 (tobacco cell line); ceRNA: competing endogenous RNA; CG: cytosine-guanine (methylation context); CHD3: chromodomain helicase DNA-binding protein 3; CHG: cytosine-histidine-guanine (methylation context); CHH: cytosine-histidine-histidine (methylation context); circRNA: circular RNA; CME: cold memory element; *CMT2*/*3*: chromomethyltransferase 2/3; *COOLAIR*: cold-induced antisense long non-coding RNA; *COR*: cold-regulated; CRISPR: clustered regularly interspaced short palindromic repeats; *CUL4*: cullin 4; ICK1: inhibitor of cyclin-dependent kinase 1; *LAZY1*: lazy plant 1; lncRNA: long non-coding RNA; *MaSPL4*: Musa acuminata SQUAMOS PROMOTER BINDING PROTEIN-LIKE 4; *MET1*: DNA methyltransferase 1; miRNA: microRNA; miR1432: microRNA 1432; *miR156c*: microRNA 156c; *miR169*: microRNA 169; miR172: microRNA 172; *miR397*: microRNA 397; *miR528*: microRNA 528; *MtNAC80*: Medicago truncatula NAC 80; *NAT1850*: natural antisense transcript 1850; NPR3: nonexpressor of pathogenesis-related genes 3; nt: nucleotides; *OsACAs*: *Oryza sativa* autoinhibited Ca^2+^-ATPase; *OsCIPK7: Oryza sativa* CBL-interacting protein kinase 7; *OsGSTU26*: *Oryza sativa* glutathione S-transferase U26; *OsHDA706*: *Oryza sativa* histone deacetylase 706; *OsJMJ703*: *Oryza sativa* jumonji 703; *OsTMF*: *Oryza sativa* TMF; *OsTrx1*: *Oryza sativa* trithorax 1; *OsWRKY72*: *Oryza sativa* WRKY 72; *PKL*: PICKLE; SHL: short life; siRNA: small interfering RNA; *SVALKA* (SVK): lncRNA regulating *CBF1* expression; *SVALNA* (SVN): lncRNA regulating *CBF3* expression; SWI/SNF: switch/sucrose non-fermentable; *TET8*: tetraspanin 8; TOE1: target of eat 1; *ZmHaspin*: *Zea mays* Haspin; *CBF*: C-repeat binding factor; *Dof1*: DNA binding with one finger 1; *DRM1*/*DRM2*: domain-rearrangement methyltransferase 1/2; EBS: early bolting in short days; *FLC*: flowering locus C; *GCN5*: general control non-derepressible 5; H2A.Z: histone variant H2A.Z; H2Aub: histone H2A monoubiquitination; H2Bub: histone H2B monoubiquitination; H3ac: histone H3 acetylation; H3K4me3: histone H3 lysine 4 trimethylation; H3K27me3: histone H3 lysine 27 trimethylation; H3K36me3: histone H3 lysine 36 trimethylation; H3K9me2: histone H3 lysine 9 dimethylation; HAT: histone acetyltransferase; HDAC: histone deacetylase; HD2C: histone deacetylase 2C; *HOS15*: high expression of osmotically responsive gene 15; INO80: INO80 chromatin remodeling complex; ISWI: ISWI chromatin remodeling complex; *MET1b*: DNA methyltransferase 1b; NF-YA: nuclear factor Y subunit A; PPO: polyphenol oxidase; PRC1: polycomb repressive complex 1; PRC2: polycomb repressive complex 2; RdDM: RNA-directed DNA methylation; ROS: reactive oxygen species; VIN3: vernalization insensitive 3.
